# Specialist Ambulance Nurses’ Perceptions of Nursing: A Phenomenographic Study

**DOI:** 10.3390/ijerph17145018

**Published:** 2020-07-13

**Authors:** Lena Forsell, Anna Forsberg, Annika Kisch, Andreas Rantala

**Affiliations:** 1 Department of Health Sciences, Lund University, SE-221 00 Lund, Sweden; anna.forsberg@med.lu.se (A.F.); annika.kisch@med.lu.se (A.K.); andreas.rantala@med.lu.se (A.R.); 2 Emergency Department, Helsingborg General Hospital, SE-205 01 Helsingborg, Sweden; 3 Department of Cardiothoracic Surgery, Skåne University Hospital, SE-224 42 Lund, Sweden; 4 Department of Haematology, Skåne University Hospital, SE-224 42 Lund, Sweden

**Keywords:** ambulance service, nursing, phenomenographic, qualitative, EMS, emergency medical services

## Abstract

Although nursing is the main area of interest in the curriculum of the specialist ambulance nursing program in the advanced level of education, there has been reported a lack of knowledge about nursing in within the ambulance service. The aim was to explore specialist ambulance nurses’ perceptions of nursing, which were explored by employing a phenomenographic approach. The study comprises individual interviews with 19 strategically selected specialist ambulance nurses. The results showed seven descriptive categories emerged detailing the variations in how the specialist ambulance nurses perceive, understand, and conceptualize the phenomenon of nursing in the Swedish Ambulance Service. Four categories revealed the specialist ambulance nurses’ qualitatively different perceptions of nursing, i.e., their role and responsibility, while three showed perceived barriers to assuming their role and responsibility, comprising culture and leadership, conditions, and framework. The seven categories are outlined in the outcome space. In conclusion, there is a very wide variety of perceptions of ambulance nursing within the Swedish Ambulance Service. There is a need for implement the nursing process both in the Specialist Nursing Pre-hospital Emergency Care education curriculum and within clinical practice. Further, there is a necessity to develop and implement nursing guidelines in the ambulance.

## 1. Introduction

Health care provided by ambulance professionals is often associated with emergency medicine, traumatology and disaster medicine [1], despite the fact that approximately 50% of assignments are labeled non-urgent [2]. The rationale behind this study is the need to explore the specialist ambulance nurses’ (SANs’) perceptions of nursing based on their experience within the Swedish Ambulance Service (AS) due to the reported lack of knowledge about nursing in ambulance care [1,3,4,5]. Recent research in the AS context reveals little or no focus on nursing [6,7]. Research pertaining to (SAN) has focused on what a SAN needs to know from educational content to critical incidents [8] and on aspects of competence and education [9].

Nursing is the main area of interest in the curriculum for both the nursing program and the specialist nursing program in the advanced level education. Thus, nursing is the specific competence for which nurses are responsible, regardless of context [10]. Nursing is both central and essential to the delivery of high-quality care in different healthcare settings [11]. In hospital environments, the risk of mortality decreased the higher the number of nurses with academic degrees who were employed [12]. When encountering patients who have severe traumatic brain injuries, registered nurses (RN) in the AS with specialist qualifications increased the quality of the assessment [13].

One aspect that presumably influences the delivery of nursing within the AS is that nursing is a complex phenomenon that can be illuminated through a variety of theories and methods. Each nursing theory provides a specific view on nursing designed to help the nurse in her/his practice. However, there is general agreement that there is a caring dimension within nursing, where caring is considered an interpersonal process characterized by expert nursing, interpersonal sensitivity, and intimate relationships [14]. Knowledge of nursing values, which represent important principles of human dignity, integrity, altruism, and justice [15], is essential for nursing practice in the AS. Thus, they should serve as a framework for the SANs’ standards, professional practice, and evaluation. The articulation of core professional values may help unify the profession [15,16] and demonstrate the value of nursing to the public [15].

The basic epistemological question about how SANs perceive nursing in ambulance care remains to be answered, thus the specific aim was to explore the SAN’s perceptions of nursing. To the best of our knowledge, no available studies focus on SANs’ perceptions of nursing within the Swedish AS. Thus, there is a knowledge gap that needs to be narrowed. Our assumption is that SAN’s perception of nursing constitutes the foundation for their assessment and judgment of patient needs and preferences. It might thus form the basis for how guidelines are used and referrals made [17].

### The Context of Swedish Ambulance Service

The SANs’ nursing responsibility is basically the same as that of other nurses, although with the inherent challenge of a huge variety of assignments involving both serious road accidents and major catastrophes as well as non-urgent, complex patients with chronic conditions in a multitude of home environments, which involve a unique responsibility for care and the need for special skills [17]. The SAN is obliged to provide nursing to patients from birth to death with reference to each person’s lived experience and life world [18]. In Sweden, the SAN makes decisions based on assessment tools and guidelines developed to evaluate patients’ medical condition. To distinguish between life threatening and non-urgent conditions the Rapid Emergency Triage and Treatment System (RETTS) is used nationwide, with only a few exceptions [19,20].

Ambulance staffing varies between and sometimes also within countries [21]. Sweden has, together with a few other countries such as Finland, the Netherlands, and Belgium, decided to staff ambulances with RNs [22]. Some counties in Sweden have chosen to staff the ambulance with SANs as the highest level of competence. In Sweden, the AS is organized autonomously in each county, which means that staffing and the required level of education for ambulance nurses varies between regions. Normally, SANs work in pairs and act as a team with another RN or an emergency medical technician (EMT), where the SAN (or RN) is responsible for the quality of care [9,23]. All nurses have three years of higher education at undergraduate level comprising 180 higher education credits ECTS with a bachelor’s degree in nursing [24]. The SAN education is at an advanced level and concludes with a Postgraduate Diploma in Specialist Nursing Pre-hospital Emergency Care and, at most universities, a Master of Science Degree (60 higher education credits ECTS in Nursing/Caring Science) [22]. SAN is a protected professional title [25].

The competence description for an RN with a postgraduate diploma in specialist nursing–prehospital emergency care outlines what is unique in the SANs’ field of competence [25]. On the basis of the patient’s individual needs and sometimes complex illnesses, the SAN should independently assume responsibility for advanced nursing in an unpredictable prehospital environment, at times under stressful working conditions and with limited resources. This requires the SAN to have the necessary skills to deliver person-centered, evidence-based, equal, and accessible ambulance care [17].

## 2. Materials and Methods

A qualitative design with a phenomenographic approach was chosen, as the aim of this study was to explore specialist ambulance nurses’ perceptions of nursing, to report the variations in how SANs perceive, understand, and conceptualize the phenomenon of nursing [26,27,28]. Qualitative analysis of human phenomena always involves both description and interpretation. According to Dahlgren and Fallsberg [28], in phenomenography description is superordinate to interpretation. The objective of phenomenography is to arrive at a conceptual description. The result is a description of similarities and differences in terms of how a certain component, aspect, or both is actually conceived of.

### 2.1. Selection and Participants

Inclusion criteria were being a SAN with a Postgraduate Diploma in Specialist Nursing Pre-hospital Emergency Care and currently working as a SAN within the Swedish AS. The participants were recruited from seven different ambulance stations in the South of Sweden covering both urban and rural areas and represent a variety of work experience, gender, and age (Table 1). Consequently, they should have gained in-depth knowledge of nursing care in the AS field in line with the existing competence description [25]. The study comprises interviews with 19 strategically selected SANs—ten women and nine men—with a mean work experience as a SAN of 7.47 years (range: 0.5–14). All participants had previous work experience as RNs (Table 1).

### 2.2. Data Collection

A person responsible for education within the AS approached potential participants who met the inclusion criteria and who were willing to participate. The contact details of those who agreed to participate were handed over to two RNs who were also master students. The two RNs then arranged and helped to conduct all the interviews. The individual interviews were performed in September and October 2017 and comprised a limited number of pre-formulated questions, with most questions posed on the basis of the initial responses. All interviews were conducted at the participants’ workplace in a quiet and secluded room at the ambulance station, either immediately before or after a shift. The participants were first asked to describe their background and experience as a SAN as well as their previous experience as a general nurse. The actual interview then began with the question “When I say nursing, what comes to your mind?” followed by “Can you please tell me how you perceive nursing within the ambulance service?” Open-ended follow-up questions were posed in order to elucidate and deepen the responses. The interviews lasted between 10 and 35 min with a mean time of 22 min. The total time for all interviews was 423 min, which generated 97 transcribed pages. The interviews were audio taped and transcribed verbatim.

### 2.3. Data Analysis

**Figure 1:**
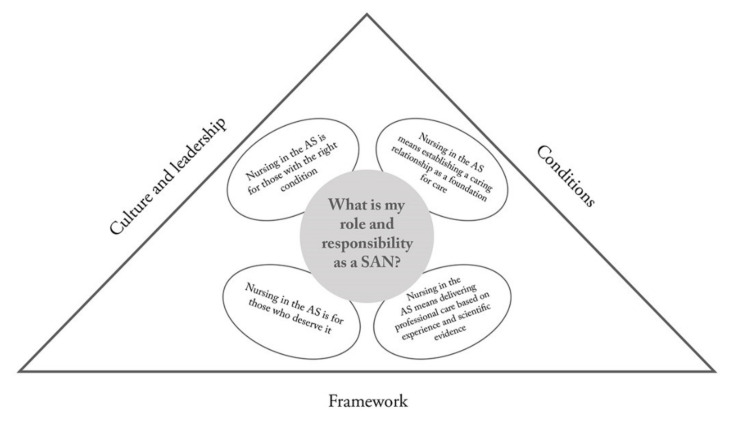
The outcome space.

All four authors participated in the subsequent analysis, which followed the seven steps described by Dahlgren and Fallsberg [28]. First, we discussed the result of the interviews with each other and read the transcribed material several times (familiarization). Second, we identified the most significant parts of the responses from each participant (condensation). In the next step, comparisons were made, and we tried to find similarities and differences in the material. In step four, the similarities and differences identified were grouped and related to each other (grouping). Table 2 and Table 3 illustrate the various perceptions behind the categories. In step five, the categories were articulated, and we decided where to draw the boundary between the different perceptions (articulating). Steps four and five were repeated several times to adjust the categories. After that we named our categories (naming) to reflect the meaning of the material. Finally, all categories were reviewed by comparing them with each other to ensure that they could not be associated with other categories (contrasting). To clarify the results in the text, important perceptions were illustrated with quotations [27]. A structure showing the relationship between the categories was designed. In phenomenography, this structure is called the outcome space and is regarded as the main result of the study [26] (Figure 1).

### 2.4. Ethical Considerations

As the study did not involve sensitive information pertaining to patients or other vulnerable persons dependent on care, ethical approval was not required in accordance with the Swedish Law concerning Ethical Review of Research Involving Humans [29]. Ethical considerations pertaining to information, consent, confidentiality, and utility were taken into account in line with the Declaration of Helsinki [30]. All participants received both verbal and written information about the study objective, that participation was voluntary, and that they could withdraw from the study at any time without any negative consequences for themselves. The participants may have found it difficult to discuss their perceptions of nursing experiences in case their views differed from those of their colleagues. However, the benefit of the study was considered higher than any harm to the participants, all of whom were clearly informed that their answers could not be traced back to them. Written informed consent was obtained by the interviewers before the interviews.

## 3. Results

Seven descriptive categories emerged, detailing the variations in how the SANs perceive, understand, and conceptualize the phenomenon of nursing in the AS. Four categories show the SANs’ qualitatively different perceptions of nursing, i.e., their role and responsibility (Table 2), while three outline the perceived barriers to assuming their role and responsibility as SANs, i.e., the culture and leadership, conditions, and framework (Table 3). The seven categories outlined in Figure 1, constitute the outcome space.

### 3.1. Nursing in the AS Means Establishing a Caring Relationship as a Foundation for Care

In this category, the informants perceived that the relational aspect is a vital part of nursing. It involved being kind and helpful, listening to the patient, holding her/his hand, establishing trust, managing the conversation, providing comfort, calming people, explaining what has happened, and what will happen next. Nursing in the AS was perceived as something that differed from hospital nursing actions/nursing actions in a hospital with different conditions and a need to take significant others into account.

“*It is all about diversity in the AS because you are in the patients’ homes. In the hospital you focus on disease, treatment, and discharging them. Here you are in their home and that includes everything*.”

(Informant no 16)

Nursing in the AS was perceived as something that is shaped by a genuine will to invite the patient into a caring relationship. The nurse is there for the patient and assisting patients was considered the SANs’ key mission in partnership with the patient and her/his significant others.

Within the caring relationship nursing was provided from a holistic perspective, mainly in the patient’s own home where the SAN perceived her/himself as a guest.

“*It is about the patient’s experience./…/to focus on the patient and provide information in an appropriate way, about the things we are about to do… We enter their bedroom, apply various strange devices and they don’t have a clue about what we are doing. That’s the moment when you need to sit down and give them your full attention, focus on their needs but also on giving them a proper explanation about what we are doing*.”

(Informant no 2.)

One possible outcome could be that patients were left at home in their familiar and safe environment, i.e., not conveyed to the Accident and Emergency Department (A&E). It was obvious that the nursing interventions had to be adjusted to the individual patient’s needs and preferences as well as her/his home situation. Based on the SAN’s impression of the living conditions and the patient’s home a comprehensive picture emerged. Aspects such as living alone, various aids, home care assistance, and the level of personal autonomy were considered. Documenting the assessment was viewed as important in order to provide a relevant foundation for the next level in the care chain. Being able to focus on one patient at the time meant less stress and simplified the assessment process. Long distance transportations provided time to establish a respectful and trusting relationship that facilitated the next encounter at the A&E after arrival at the hospital. As the caring relationship was viewed as the foundation of nursing, factors that might negatively affect it were night shifts, tiredness, own personal problems, and the significant others’ concern and anxiety.

### 3.2. Nursing in the AS Means Delivering Professional Care Based on Experience and Scientific Evidence

In this second category, it was perceived as important to emphasize the distinction between the SAN and professional nurses in other contexts, while highlighting the SAN’s status and significance. The SAN was perceived as an expert on nursing, not at all like community nurses, but completely autonomous and possessing the knowledge to manage emergency nursing. The participants’ perception was that nursing actions are shaped first and foremost by the personal character of the SAN but also by education and nursing science. It was perceived that research advances nursing practice but that it is important to narrow it down. Nursing was perceived as either possible to develop through research and formal education or impossible to develop because developing nursing is totally dependent on the personality and will of the individual SAN. This revealed an inherent paradox, as some of the participants considered that research, education, supervision, and peer learning were valid tools for the development of nursing, while others perceived nursing as a personality trait inherent in one’s nature.

“*If all the SANs in your crew are skilled in nursing you can learn from them even if you don’t possess the skills or competence yourself*.”

(Informant no 7.)

The SAN’s role and responsibility was perceived as helping the patient by being distanced and objective and not necessarily through a caring relationship. The choice of nursing actions might be affected by the SAN’s professional competency and ontological assumptions. Nursing was perceived as highly personalized and mirrored by the SAN’s actions.

### 3.3. Nursing in the AS is for Those Who Deserve It

In this category the informants described nursing as role play where the AS approaches the patient in a professional manner even if the SAN dislikes the patient.

“*That is how you behave, you put on ‘your nursing suit’*.”

(Informant no 7.)

It was clear that the quality and magnitude of nursing interventions were affected by the patient’s behavior. If the patient’s behavior was considered bad, the nursing care was limited to a minimum. This also occurred if someone was viewed as rude or under the influence of alcohol or drugs. Even if the SAN was aware of the regulations and professional standards, it was perceived as a professional right to apply zero tolerance based on one’s own personal level of moral stance.

“*Because if we meet someone who has a bad manner, it affects how we provide nursing as they simply don’t receive any care. You see, personally I do all I can for the patient, but if they are real scumbags or if they do things to you, they get as little care as possible. You give basic treatment for their symptoms and nothing more. Of course, there are regulations and I’m obliged to perform certain nursing interventions, but there is a limit to what I tolerate. I apply zero tolerance. You simply ignore the patient’s wellbeing and merely do the basics*.”

(Informant no 7.)

### 3.4. Nursing in the AS Is for Those with the Right Condition

In this category, the SANs’ perception of nursing was the provision of biomedical care to the patient. The patients were viewed as an object, a body in need of biomedical treatment. If there was an emergency medical condition the patient was prioritized and received medical treatment. Older patients were considered vulnerable and entitled to more nursing interventions than younger ones. In this category, nursing is influenced by the SAN’s personality and her/his personal method of providing nursing. The patient must qualify for ambulance care by her/his behavior and by having the “right” medical condition (i.e., not patients presenting with, e.g., psychiatric symptoms). If the patient fits the profile and criteria for ambulance care she/he is entitled to good nursing. However, the SAN’s empathy and patience are limited if the patient is deemed to be in need of a community nurse or care from the primary health care center.

“*Some of the patients we visit could instead have been taken care of by a community nurse or the home care service, or they could have gone to their primary healthcare center. And there is a lot of stress and you get annoyed and perhaps you don’t care as much as you should about the patient…. well you might care, but you don’t have the same patience… when there is no food break on your shift or time to go to the toilet, and you constantly visit patients who could have gone by ordinary transport to the hospital or whatever.. /…/I can’t forbid people to call for an ambulance, of course they are allowed to do that, but sometimes it is clear that we are not the ones to solve their problems as they could do so on their own*.”

(Informant no 6.)

There was a perception that the SAN uses fewer friendly words and ignores certain things when the patient has the “wrong” condition. This also occurred during nightshifts and if the team spirit was poor.

“*I believe that you lose your temper much more easily, you don’t spend that much energy…. you might try to solve the patient’s problem by helping her/him to the primary care center or manage to wait for the community nurse…But you feel frustrated and you don’t provide the same nursing care and you are less empathic*.”

(Informant no 6.)

### 3.5. Culture and Leadership as Barriers for Nursing in the AS

In this category, the various perceptions of the culture and leadership stemmed from a sense of inferiority, revealing that nursing has low priority and that managers determine the status of nursing in the AS. There is a perception that nursing is something that is not performed in the AS.

“*And ehh...if you choose to work in the ambulance service it’s sort of expected that you should almost hate nursing as well*.”

(Informant no 17.)

There were also perceptions that the organization affects nursing in different ways. If the management has a bad relationship with staff, the staff members become frustrated, which influences their relationship with the patient and significant others. However, when staff members have confidence in management they respond better to patients and their significant others.

“*But if you want the staff to function well, it is important that the management is first and foremost nice, that you can trust them. Because that’s what I feel I’m suffering from in this business. You never really know where you are. You don’t know if they are honest*.”

(Informant no 12.)

The team in which the SAN works also has a great impact on nursing. If the team has a negative attitude towards nursing it characterizes the SANs´ work and can also affect the choices made for the patient. For example, some patient groups are not expected to be transported by ambulance for further care and nursing.

“*You are never expected to bring a back pain, for example, to the A & E because they do nothing*.”

(Informant no 15.)

There was also a perception that SANs do not discuss nursing. Only the medical treatment, what was done, and what effects it had on the patient are discussed to some extent. As nursing does not have a high status/is not prioritized the SANs do not talk about it.

### 3.6. Conditions as a Barrier for Nursing in the AS

In this category, all the perceptions of barriers regarding conditions were based on acceptance of the fact that the AS is not organized for the provision of nursing.

“*We also have no good tools, you can always convey the patient based on your own impression /…/but we have no good tools for nursing*.”

(Informant no 2.)

The medical work is always number one. The provision of nursing depends on the time available, the perceived vulnerability and age of the patient, as well as her/his attitude and conduct. One perception concerned the fact that AS nursing guidelines could promote development by facilitating quality evaluation. However, the current lack of such guidelines was perceived as a barrier along with insufficient records of the patient’s nursing needs and lack of equipment for specific nursing interventions, e.g., wound dressing and changing catheters.

“*I think it’s easier for it (nursing) to fail than medication and suchlike. You cannot review it in the same way as reviewing a medical record*.”

(Informant no 12.)

The category conditions included various perceptions that empathy varies depending on the time of the day and type of assignment. Working night shifts and being tired were perceived as factors that have a negative effect on how nursing care provided and could also justify the decision to ignore the patient. Finally, it was perceived that the longer one’s work experience the bitterer one becomes.

### 3.7. Framework as a Barrier for Nursing in the AS

The AS is regulated by guidelines, e.g., the RETTS, and in this last category, perceptions varied regarding the value of these algorithms that solely focus on the disease as evidenced by the medical signs and objectively verified symptoms. There were perceptions that the RETTS does not support nursing but is performed for someone else’s sake. There was also a perception that the RETTS is the most important thing that has happened in the AS due to the fact that patients are now being assessed objectively and not judged on personal interpretations and opinions about who is in need of care. The ambulance record focuses solely on documenting the correct triage level based on RETTS as well as the patient’s visual signs and vital parameters with no space to document nursing.

“*There are also check boxes where you can fill in the patient’s nursing needs, if the patient needs help with walking and standing or eating and drinking and suchlike*”.

(Informant no 15.)

There are perceptions that the lack of nursing guidelines affects the care of the persons in need of nursing and also that the SAN education as well as the training provided in the workplace do not focus on nursing, but mostly on medicine and practical issues.

“*You should have at least four years of nursing in...yes…the basic education and the specialist training… then you should be a little better at understanding this and be a little more interested in nursing*.”

(Informant no 15.)

### 3.8. Outcome Space

The outcome space (Figure 1) illustrates the relationship between the seven different categories, where the question: “What is nursing in ambulance care?” is answered based on four qualitatively different descriptions of the SANs’ role and responsibility. These four descriptions reflect the fact that there are barriers to what nursing is and how it can be performed. These barriers form three different descriptive categories, constituting the boundaries of the SANs’ perceived role and responsibility. The similarities found between the four descriptive categories pertaining to the role and responsibility of the SAN were that the nursing role was perceived as associated with the personality of the nurse and subsequently that nursing was provided based on the nurse’s personality. The nursing spirit was emphasized as the core of the professional AS. Either one has or does not have the spirit or interest in the patient, therefore nursing was considered something inherent that cannot be learned. Providing a service and ensuring that patients are transported to the right level of care was deemed essential, as was making sure that the patient is well when it is decided not to transfer her/him to the hospital. Nursing in the AS was also perceived as different from the nursing provided in a hospital, often described as something instrumental and concrete such as inserting a venous catheter and administering medication IV or positioning the patient on the stretcher and preventing injuries during transportation.

## 4. Discussion

The key findings in this study was that four qualitatively different roles of the SAN were identified and that these different ways of approaching the patients leads to consequences in terms of how the patients are assessed and different care delivered. The informants’ perceptions of nursing in the AS were clearly generated from their perceptions of general nursing and they related their perceptions to their previous experience of being an RN in a hospital ward. Interestingly, general nursing was perceived as multifaceted and more differentiated than nursing in the AS. The SANs considered that nursing was mainly something performed in hospital wards and not so much in the AS.

It is often a major decision for patients to call an ambulance, and quite frequently persons other than the patient her/himself are involved in the decision-making process [31]. How nursing care is provided is determined by the competence and attitude of the SAN in addition to her/his perspective on the human being. The starting point for good nursing rests on the professional’s expectation of the importance of the caring relationship [32]. In the results of our study, only the first two descriptive categories revealed that SANs were able to invite the patient into a caring relationship. In the two latter categories, the caring relationship was subject to conditions that were up to the patient to overcome by behaving properly or having the right illness. The nursing situation is defined and based on patient behavior, the nurse’s reaction and action, where the immediate response is unique to each situation, and crucial for understanding the meaning of the patient’s behavior. Orlando’s [33] reflective nursing process reveals the relationship between patient and nurse, where the nurse’s actions affect the patient and vice versa. Our findings clearly show that how the SAN acts and reacts is based on the patient’s behavior, which confirms Orlando’s theory.

The findings suggest that care provided by each of the four qualitatively different professional SAN approaches can vary, which might have consequences in terms of assessment, decision-making, treatment, and referral/non-referral to the hospital. In this study, it can be argued that the existing guidelines promote an attitude where patients who have the right condition should be given access to ambulance care. Can one therefore blame the two types of SAN who prioritize based on the patient’s behavior and treatment as well as condition? The informants perceived that the organization and the framework did not support the practice of nursing. Nevertheless, there was a group of SANs who were clearly caring and strived to establish good care relationships as a basis for the nursing network. They succeeded in maintaining the moral bond of nursing despite poor conditions. If the context evolved to embrace nursing based on caring relationships, perhaps more SANs could develop their professional skills and thus meet the public’s expectation of being taken seriously. The role of the SAN originates from being a member of a transport organization that provides basic care during transport from the patient’s home to the hospital, despite the fact that the AS has moved from a traditional masculine “fire brigade culture” to a more egalitarian and gender balanced “healthcare culture” [23]. To meet the diversity of patients’ needs and preferences within the AS, the organization must be prepared for and allow SANs to practice nursing care, as failure to do so will result in the emergence of a culture with a strong medical view of patients and absence of relevant interventions.

When the ambulance arrives at the scene the patient may feel relieved that help is at hand, especially if she/he made the decision to phone [31]. All patients expect to be taken seriously and treated with respect. When not taken seriously, the patient may doubt her/his own judgment and feel guilty and ashamed about bothering the AS [34]. Being treated disrespectfully and having to prove oneself worthy of care can adversely affect both present and future care. However, if the encounter is caring and the SAN responds to the patient and builds a partnership, the patient dares to surrender into the hands of the SAN, thus gaining increased confidence in the AS and healthcare in general.

Some patients do not expect to meet a SAN who makes an assessment, but only fast transportation to a hospital. If such patients encounter a SAN who adopts the approach “Nursing in the AS means establishing a caring relationship as a foundation for care” or “Nursing in the AS means delivering professional care based on experience and scientific evidence” the prerequisites for a good meeting are in place and the nursing process is likely to be handled correctly. However, if the patient instead encounters a SAN who holds the view that “Nursing in the AS is for those who deserve it” or “Nursing in the AS is for those with the right condition” she/he has to qualify by having the “correct” diagnosis and possibly also behave in the “right” way for care. Patients who meet these requirements are entitled to good nursing. Some parallels can be drawn with Schuster [32], who identified three models for how nurses constitute themselves in the meeting with seriously ill patients. The “Method-Oriented Nurse” believes that she/he knows what the patient’s needs are and has a narrow perception of nursing focused on actions, which leads to the risk of the patient becoming a passive object. The “Neutral Nurse” distances her/himself from the patient by entering into a professional role, while the “Good Nurse” is characterized by the image of the nurse as a helper and donor. The “Good Nurse” is also altruistic and loves being the driving force. Based on the findings in our study, we argue that in order to provide good nursing care the SAN should see the whole person, be open, reflective, and focus on how illness is experienced by patients [35]. Furthermore, there is a need for an organizational improvement of the AS, including the development of holistic guidelines covering both the perspective of disease and illness and providing medical and nursing interventions.

Based on the findings, there is also room for a change in the curriculum of the Specialist Nursing Pre-hospital Emergency Care education. Nursing and nursing values are developed through education [36], which is also reflected in the SANs’ perceptions. One explanation for the almost dialectic way the SANs consider nursing in the AS can be an imbalance between medical, nursing, and contextual knowledge in the SAN education, where the main focus is on medical knowledge and the least on nursing [22]. In the study by Sjölin et al. [22], data were generated from 49 nursing and medical science courses in specialist nursing programs in prehospital care from different Universities in Sweden. Each university organizes its educational content into courses. The course content was described as medical, nursing, and contextual knowledge, with the main focus on medical knowledge and the least on nursing knowledge, despite the latter being the SANs’ area of competence. The Specialist Nursing Pre-hospital Emergency Care education consists of theoretical courses and clinical practice. The latter is important for acquiring the skills required to work professionally as a SAN. However, personal chemistry as well as the supervisor’s attitude and view of the work are factors that may affect the learning situation [37]. Treatment guidelines and the SAN education are based on the fact that the patients the SAN will encounter are critically ill or injured in accordance with a medical definition that does not take account of the patient’s own experience of illness. However, Hörberg, Lindström, Kalen, Scheja, and Vicente [38] described that nurses new to the AS felt well prepared to handle trauma and acute illness, but that the majority of the patients they encountered did not fit this template and that the guidelines did not cover all aspects of ambulance care.

In the present study, the SANs reported that barriers to nursing practice were the impression that they have no nursing guidelines to follow and that the nursing content in educational programs is limited. This is consistent with the study by Rosén, Persson, Rantala, and Behm [4] that explored the AS as experienced by current and former employees. The participants in the above-mentioned study also perceived that the AS guidelines were derived from a medical perspective and that the AS organization did not create the right prerequisites for the care they provide. The treatment guidelines focus on acute assignments, which in reality only constitute a small part of their work. It is obvious that SANs would benefit from clearer guidelines that include nursing aspects.

### Strengths and Limitations

By using the phenomenographic method we gained knowledge about the variations in the SANs’ perceptions of nursing based on their work experience in the AS. In phenomenography the focus is on differences in human phenomena and the aim is to describe these differences in terms of the conception of the surrounding world [28]. Accordingly, the outcome categories from a phenomenographic analysis constitute peoples’ various ways of thinking about their experiences [27]. The outcome space is “born” in the moment that the researcher reads the narratives, thus it grows out of the empirical interview material. The category system is the product of the researcher’s analysis of a particular material.

Phenomenography was developed in the field of education (where learning is seen as a change in the learner’s ability to experience a phenomenon) to answer certain questions about thinking and learning [26]. Therefore, the results of phenomenographic studies have implications for education, but have also been found to be useful in nursing research [27], which we have demonstrated and argued in this study.

Regarding methodological quality, this study adheres to the four quality criteria for ensuring trustworthiness in qualitative research developed by Lincoln and Guba [39]. The relatively short duration of the interviews is a possible threat to credibility. No new perceptions emerged after eleven interviews, which agrees with Larsson and Knutsson-Holmström [40], who stated that new perceptions rarely emerge after 10–12 interviews. However, to ensure sufficient data and to compensate for some of the shorter interviews we performed in a total 19 interviews. Longer interviews might have increased the credibility of the study, but the number of different perceptions that emerged shows that the data were considered sufficient. The presentation of all these perceptions enhances the transparency of the analysis. While dependability may have been influenced by the fact that a person responsible for education within the AS context approached the potential participants, the varied and qualitatively different perceptions indicate that this did not constitute a limitation. Confirmability was ensured as the participants described perceptions of nursing in the AS context, which corresponded to the aim of the study, thus providing rich and relevant data that were considered useful. The profound clinical pre-understanding of two of the authors gained from working as SANs in the AS for many years could have had an impact on the interpretation and analysis. However, they did not work together with the study participants. Furthermore, there was an open dialogue with the second and the third author who have no clinical experience of this context but extensive knowledge of qualitative research, which helped reduce potential misinterpretation and ensured the confirmability and trustworthiness of the study. Finally, transferability can be considered high as the participants represent catchment areas comprising both rural and urban settings, as well as diversity in terms of socio-economic status. However, perceptions of nursing in the AS are most likely dependent on factors such as the workplace culture and the attitude of management, thus limiting transferability to contexts that differ from the specific ambulance context in the study.

## 5. Conclusions

There is a very wide variety of perceptions of ambulance nursing within the Swedish AS. Seven descriptive categories emerged, detailing the variations in how the SANs perceive, understand, and conceptualize the phenomenon of nursing in the AS. Four qualitatively different professional SAN approaches exist in ambulance care, presumably affecting the assessment and judgment of the patients’ condition, while three categories outline the perceived barriers to assuming their role and responsibility as SANs. An in-depth understanding of SANs’ perceptions of their nursing experiences is essential in order to properly implement the nursing process both in the Specialist Nursing Pre-hospital Emergency Care education curriculum and within clinical practice. There is a need for the development and implementation of nursing guidelines in the AS.

These findings raise questions about the role of SANs in the Swedish AS and the consequences of the four different professional profiles. The results suggest that SANs’ knowledge of what nursing is and what it means to the patient is somewhat lacking. The results should have an impact on the in future development of the SAN educational curriculum. Knowledge of general nursing and in particular contextual nursing should be highlighted and strengthened both in the education to become a SAN and also in AS internal training. We recommend professional meetings in each AS setting in order to reflect on, process, and delineate the essence of nursing and its implications for nursing practice within the AS with the help of the findings of this study.

## Figures and Tables

**Table 1:** Characteristics of the specialist ambulance nurses (SANs) (n = 19).

**Years of Age**	**Men 47% (*n* = 9)**	**Women 53% (*n* = 10)**
<30	1	1
31–40	2	5
41–50	5	3
51–60	1	1
>61	−	−

**Table 2:** The SANs’ perceptions of what nursing means and how it affects their perception of their role and responsibilities.

Categories	Variation in Perceptions of the SAN’s Role and Responsible
Nursing in the AS means establishing a caring relationship as a foundation for care	Nursing means: - establishing a relationship and focusing on the patient - caring for patients who are unable to take care of themselves - emotions, empathy, and understanding the patient - everything we do to the patient, physically, and emotionally - providing comfort and information - how we touch patients and place them on the stretcher - things you do with your hands and soul, without technical devices
Nursing in the AS means delivering professional care based on experience and scientific evidence	Nursing means: - meeting basic needs - helping the patient with her/his needs - assessing signs and symptoms - interventions aimed at health promotion - the conversation - non-pharmacological pain relief
Nursing in the AS is for those who deserve it	Nursing means: - the view of the person affects nursing - the attitudes of patients and relatives influence how nursing is provided - patients with a bad manner receive little or no nursing care - patient groups deemed vulnerable by the SAN receive more nursing care
Nursing in the AS is for those with the right condition	Nursing means: - the level of emergency is decisive for whether nursing is provided - emergency and non-emergency nursing, where a psychiatric problem is not deemed an acute condition - the delivery of nursing care differs between young and old patients - frequent callers receive the worst nursing care and things are omitted

**Table 3:** The SANs’ perceptions of the Ambulance Service context and organization affecting their perceived role and responsibilities.

Categories	Variation in Perceptions
Culture and leadership as barriers for nursing in the AS	- The employer’s perspective on nursing means a great deal - The leadership, environment, and workplace are decisive for nursing practice - You are supposed to hate nursing when you work in the ambulance - Nobody wants to develop nursing and nobody cares - There is no status in nursing	
Conditions as a barrier for nursing in the AS	- Enough time ensures excellent nursing care - The medical work is always the priority - The strong medical focus is a barrier to nursing care - We lack tools for nursing - We cannot perform practical interventions - We cannot perform wound dressing or flush catheters - It is impossible to record nursing in the ambulance journals - You are unable to follow-up your patients - Working night shifts and being tired have a negative effect on how you deliver nursing care - Empathy varies depending on the time of the day and type of assignment - The longer your work experience the more bitter you become	
